# Wave Propagation of Junctional Remodeling in Collective Cell Movement of Epithelial Tissue: Numerical Simulation Study

**DOI:** 10.3389/fcell.2017.00066

**Published:** 2017-07-19

**Authors:** Tetsuya Hiraiwa, Erina Kuranaga, Tatsuo Shibata

**Affiliations:** ^1^Department of Physics, Graduate School of Science, University of Tokyo Tokyo, Japan; ^2^Laboratory of Histogenetic Dynamics, Graduate School of Life Sciences, Tohoku University Sendai, Japan; ^3^Laboratory for Histogenetic Dynamics, RIKEN Center for Developmental Biology Kobe, Japan; ^4^Laboratory for Physical Biology, RIKEN Quantitative Biology Center Kobe, Japan

**Keywords:** mathematical model, vertex model, mechanobiology, collective cell migration, epithelial cells, cell intercalation

## Abstract

During animal development, epithelial cells forming a monolayer sheet move collectively to achieve the morphogenesis of epithelial tissues. One driving mechanism of such collective cell movement is junctional remodeling, which is found in the process of clockwise rotation of *Drosophila* male terminalia during metamorphosis. However, it still remains unknown how the motions of cells are spatiotemporally organized for collective movement by this mechanism. Since these moving cells undergo elastic deformations, the influence of junctional remodeling may mechanically propagate among them, leading to spatiotemporal pattern formations. Here, using a numerical cellular vertex model, we found that the junctional remodeling in collective cell movement exhibits spatiotemporal self-organization without requiring spatial patterns of molecular signaling activity. The junctional remodeling propagates as a wave in a specific direction with a much faster speed than that of cell movement. Such propagation occurs in both the absence and presence of fluctuations in the contraction of cell boundaries.

## Introduction

The morphogenesis of embryonic development involves epithelial tissue deformations (Tomer et al., 2012). Epithelial tissue consists of epithelial cells that adhere to each other through cell-cell junctions, such as adherence junctions and tight or septate junctions. These intercellular junctions are connected to the intracellular actin cables of individual cells (Harris and Tepass, 2010; Takeichi, 2014), which generate a mechanical tension that is exerted on the cell-cell junction. The mechanical forces of an individual cell are further transmitted to its neighboring cells through the junctions. Consequently, the cell monolayer achieves mechanical integrity as an epithelial tissue. While maintaining the balance of mechanical forces in the tissue, an epithelial sheet can dynamically deform to elongate and fold during development. Such epithelial deformations are often achieved through the positional rearrangements of epithelial cells (Bertet et al., 2004; Nishimura et al., 2012; Tomer et al., 2012). Such cell rearrangements in a cell sheet occur through the extension and contraction of cell-cell junctions in the apical plane, which lead to junction exchange between neighboring cells, which is called a T1 transition, while maintaining the monolayer integrity (Bertet et al., 2004; Blankenship et al., 2006; Rauzi et al., 2008). Therefore, the remodeling of the network of cell-cell junctions in the apical plane is a key mechanism by which epithelial tissue achieves a large deformation.

A typical example of a deformation driven by the junctional remodeling is convergent extension, by which a tissue elongates in one direction while shrinking in the perpendicular direction (Tada and Heisenberg, 2012; Collinet et al., 2015). During this process, the cell junctions perpendicular to the extension direction shrink and cells intercalate with each other along the direction of tissue convergence (Munro and Odell, 2002a,b). Consequently, the tissue achieves a large deformation without changing the cell size or shape and thus the internal stress. A well-studied example is the germ band extension (GBE) occurring during *Drosophila* early embryogenesis. During GBE, epithelial cells undergo intercalation directed along the dorso-ventral (DV) axis. A high accumulation of non-muscle myosin II (Myo-II) at the anterior and posterior cell boundaries increases the strength of the junctional contraction, causing a decrease in junctional length. These DV-oriented cell intercalations cause the tissue to narrow along the DV axis and lengthen along the anteroposterior (AP) axis, resulting in GBE. In other examples of large scale morphogenesis driven by the junctional remodeling, mediolaterally oriented cell intercalation contributes to kidney tubule elongation in *Xenopus* (Lienkamp et al., 2012), and polarized apical cell constriction drives neural tube invagination in the chick (Nishimura et al., 2012).

Such cell intercalation accompanied by the junctional remodeling is also a driving mechanism for the collective cell movement of epithelial tissue. During the morphogenesis of *Drosophila* male terminalia, the genitalia undergo a 360° clockwise rotation, which induces dextral spermiduct looping (Suzanne et al., 2010; Kuranaga et al., 2011). The *Drosophila* genitalia rotation (DGR) is achieved by the collective clockwise movement of surrounding epithelial cells. We previously reported that this collective cell movement is driven by polarized cell intercalation at the right oblique cell boundaries in the surrounding epithelial tissue (Sato et al., 2015a). The moving cells intercalate while remaining attached to their neighboring cells. Most of the remodeled cell boundaries form right oblique angles with the AP axis and show Myo-II accumulation. In addition, numerical simulations revealed that such diagonally polarized cell intercalation is sufficient to induce unidirectional cellular movement (Sato et al., 2015a). We also revealed that such left-right asymmetry of the cell boundary motion accompanied by AP asymmetry of the tissue is indispensable for the unidirectional movement (Sato et al., 2015b). Since epithelial cells also have the asymmetry of apico-basal polarity, the left-right asymmetry of Myo-II accumulation and resultant cell boundary motions in the planer plane can be referred to as the chirality, or the handedness, of the cells (Sato et al., 2015b).

Both GBE and DGR are induced by the cell intercalation. However, the dynamicity of cells in the tissues shows a strong contrast between the two situations. GBE is induced by a tissue deformation involving different aspect ratios of singly rearranged cells. In contrast, DGR involves the movement of a cell collective. Specifically, small cells of about 5-μm diameter move a distance of 300 μm or more in over 12 hours (Kuranaga, 2012; Sato et al., 2015a). What makes this difference in the dynamicity of the two systems? One obvious difference is the direction of cell intercalation; it is perpendicular to the direction of cell movement in GBE (Collinet et al., 2015), while diagonal in DGR (see Figure 4 in Sato et al., 2015a). Consequently, the cell intercalation events take place transiently in GBE until the tissue deformation finishes, while in DGR the cell intercalation events can continue to occur. However, this diagonal direction of cell intercalation is not sufficient to explain the collective cell movement that lasts for more than 12 hours. One factor underlying the difference in these processes is predicted to be the spatiotemporal dynamics (time order and distribution) of the cell intercalation. What determines the spatiotemporal dynamics of the cell intercalations?

A molecular signaling activity can regulate the spatiotemporal dynamics of junctional remodeling and cell rearrangement. But, it can also be organized spontaneously through mechanical processes. Here, we referred to such a spontaneous spatiotemporal organization of cell intercalation without relying on molecular signaling activity as self-organization. After a certain period of time, a series of T1 transitions gives a viscous property or plasticity to a tissue that enables large-scale deformation. In contrast, in the shorter term, elastic behavior can appear in response to the T1 transition of an individual cell that is induced by intracellular molecular signaling. When individual T1 transition is induced, mechanical forces are exerted on the surrounding cells. Such forces can be transmitted through the network of cell-cell junctions. The transmission of such a force usually ceases within the distance of a few cells (Farhadifar et al., 2007). If it further triggers another T1 transition, however, the transmission can occur over a longer distance. Consequently, the junctional remodeling and cell rearrangement could spread throughout an entire tissue in a spatiotemporally correlated way. It is therefore possible that the large scale reorganization of epithelial tissue is not only instructed by molecular signaling activity of axis information, but also involves self-organization through mechanical coupling among cells. However, such self-organized spatiotemporal dynamics of cell intercalations have not been described in our previous study. Therefore, the question that we address in this paper is whether the transmission of mechanical forces due to the cell intercalation can occur without spatiotemporal signal instruction, and how the cell rearrangements by junction remodeling are spatiotemporally organized during the collective epithelial cell movement in DGR.

In this paper, we investigated theoretically the spatiotemporal dynamics of cell movement in the ring-like epithelial tissue that surrounds the male genitalia, using a mathematical model that we described previously (Sato et al., 2015a,b). We show for the first time that the T1 transitions and cell rearrangement propagate in space in the same direction of the collective cell movement, leading to the propagation of cell velocity change in space with speed faster than the collective cell movement.

## Model and methods

### Numerical model of the epithelial cell monolayer with left–right cell asymmetry

**Figure 1:**
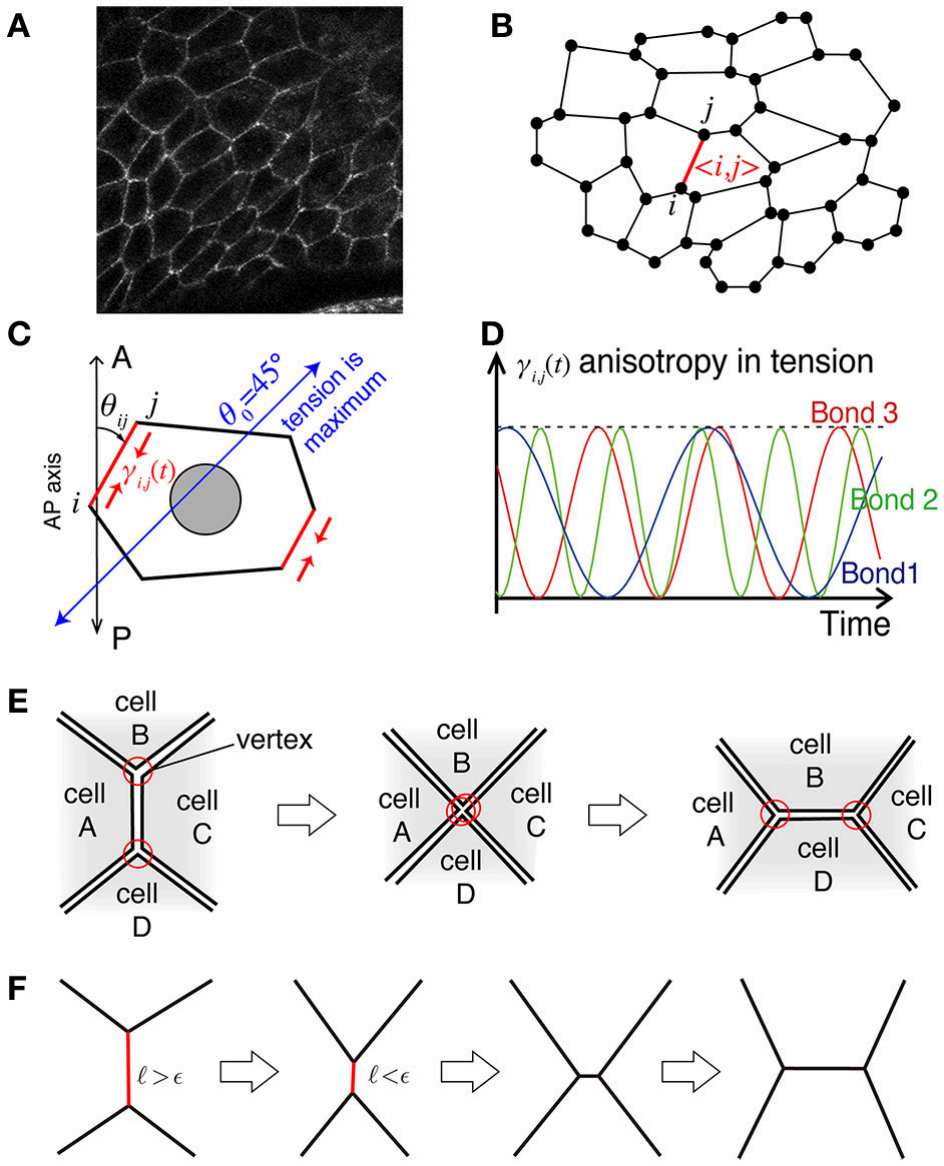
Illustration of the mathematical model. **(A)** Actual cell–cell junctions visualized with E-cadherin. The epithelial cell monolayer resembles polygons whose shared sides correspond to cell–cell junctions. **(B)** The cell vertex model in two dimensions, modeling the polygon-like structure of the network of cell–cell junctions. The vertex index is indicated by *i* and *j*, and the bond index is given by 〈*i, j*〉. **(C)** Bond-dependent tension strength. When the bond is tilted in the clockwise direction by $\theta_{0}={4.html5}^{{}^{\circ}}$ from the AP axis, the line tension of the bond is maximum. **(D)** The heterogeneity in line tension strength in the model. **(E)** Process of junctional remodeling, called a T1 transition. Lines indicate the edges of cells. **(F)** T1 transition process in the numerical simulation.

We describe the model for the rotational motion of *Drosophila* genital disc that we have introduced in the previous work (Sato et al., 2015a). We use a vertex cell model in two-dimensional space to simulate the in-plane motions of cells in an epithelial monolayer tissue (Nagai and Honda, 2001; Farhadifar et al., 2007). When we look at the epithelial cell monolayer from the apical side (Figure 1A), individual cells exhibit a polygonal shape. Based on this observation, the cell shapes are described as polygons with vertices and edges (Figure 1B). A polygon in the model is specified by the location of the *i* th vertex, **r**_*i*_ = (*x*_*i*_, *y*_*i*_), and the bond 〈*kl*〉 that connects vertices *k* and *l*. By considering the force balance between the potential force −∂*E*({**r**_*i*_}_*i*_, {γ_*kl*_}_〈*kl*〉_)/∂**r**_*i*_ and the friction force of the simplest form −η_*i*_**r**_*i*_/*dt*, the time evolution equation of the vertex model is given by

(1) $$\begin{array}{l} \frac{d{\text{r}}_{i}}{dt}=-\frac{1}{\eta_{i}}{\frac{\partial E\left({\left\{{\text{r}}_{i}\right\}}_{i},{\left\{\gamma_{kl}\right\}}_{\langle kl\rangle }\right)}{\partial {\text{r}}_{i}}|}_{\gamma_{kl}\;=\;\hat{\gamma }\left(\theta_{kl}\right)} \end{array}$$

Here, the potential function *E* is given by

(2) $$\begin{array}{l} E\left({\left\{r_{i}\right\}}_{i},{\left\{\gamma_{kl}\right\}}_{\langle kl\rangle }\right)=E_{\text{p}}+E_{\text{t}}+E_{\text{b}}. \end{array}$$

The first term on the right hand side of Equation (2), *E*_p_, is the contribution from the hydrostatic pressure within a single cell given by

(3) $$\begin{array}{l} E_{\text{p}}=\frac{K_{a}}{2}\overset{N}{\underset{\alpha =1}{\sum }}{\left(A_{\alpha }/A_{0}-1\right)}^{2}, \end{array}$$

with the actual area *A*_α_ of the α-th cell, the preferred area *A*_0_, and the coefficient *K*_*a*_ controlling the strength of the pressure. The integer *N* indicates the total number of cells. The second term *E*_t_ is the potential function for the total tension on cell-cell adhesions, given by

(4) $$\begin{array}{l} E_{\text{t}}=\frac{K_{p}}{2}\overset{N}{\underset{\alpha =1}{\sum }}{\left(L_{\alpha }/L_{0}-1\right)}^{2}+\underset{\langle ij\rangle }{\sum }\gamma_{ij}\left(t\right)\ell_{ij}. \end{array}$$

The first term is the contribution due to the mismatch between the actual perimeter *L*_α_ of the α-th cell and the preferred perimeter *L*_0_, with the coefficient *K*_*p*_ controlling the tension strength. The second term is another contribution due to the cell's spontaneous and bond-specific transport of molecules, such as myosin II and cadherin, which exert tension with the bond-specific tension strength γ_*kl*_ (Figure 1C), which can be time dependent, as described in detail below.

The last term *E*_b_ is the potential function describing the boundaries of the system as explained below. The epithelial monolayer forms a ring-like tissue. It is flanked by two circular walls with different radii (*R*_out_ and *R*_in_ for the outer and inner walls, respectively), both of which are centered at the origin (0, 0). These walls mimic the concentric ring-like tissue structure that surrounds the *Drosophila* male genitalia at the larval stage (Sato et al., 2015a). We assume that additional forces are exerted on the vertices *i* located along the outer wall (i∈O) and the inner wall (*i* ∈ *J*), described by the potential function as

(5) $$\begin{array}{l} E_{\text{b}}=\frac{k_{\text{out}}}{2}\underset{i\in O}{\sum }{\left(r_{i}-R_{\text{out}}\right)}^{2}+\frac{k_{\text{in}}}{2}\underset{i\in J}{\sum }{\left(r_{i}-R_{\text{in}}\right)}^{2}, \end{array}$$

where $r_{i}=\sqrt{x_{i}^{2}+y_{i}^{2}}$ is the distance of the *i* th vertex from the origin (0,0), and *k*_out_ and *k*_in_ are coefficients. We also assume that the friction coefficients η_*i*_ are different between vertices along the outer and inner walls.

The bond-specific tension γ_*kl*_ includes regulatory processes that are cell-autonomous and not described by potential functions. We thus consider that the force in Equation (1) is given as a derivative with respect to the position **r**_*i*_ under the condition that γ_*kl*_ is independent of **r**_*i*_. After deriving the force, we consider that the tension strength γ_*kl*_ depends on the direction θ_*kl*_ of the bond 〈*kl*〉 around the AP axis of the tissue, as $\gamma_{kl}\left(t\right)=\hat{\gamma }\left[\theta_{kl}\left({\text{r}}_{k},{\text{r}}_{l}\right)\right]$. This consideration breaks the conservation of the potential *E* in Equation (2), and keeps the system from relaxing to the equilibrium state (Sato et al., 2015b). The specific form of γ_*kl*_(*t*) in this article is given by

(6) $$\begin{array}{l} \gamma_{kl}\left(t\right)=\gamma_{c}\text{co}{\text{s}}^{2}\left(\theta_{kl}\left(t\right)-\theta_{0}\right), \end{array}$$

where $\theta_{0}=+45{}^{\circ}$ and γ_*c*_ is a constant giving the maximum tension. We define the sign of the angle θ_*kl*_ in the clockwise direction around the AP axis. We also consider the case in which temporal fluctuations are present in the tension γ_*kl*_(*t*), given by

(7) $$\begin{array}{l} \gamma_{kl}\left(t\right)=\gamma_{c}\text{co}{\text{s}}^{2}\left(2\pi f_{kl}t+\delta_{kl}\right)\text{co}{\text{s}}^{2}\left(\theta_{kl}\left(t\right)-\theta_{0}\right), \end{array}$$

where *f*_*kl*_ is the frequency and δ_*kl*_ is the initial phase of the bond 〈*kl*〉. The frequency and initial phase are given by uniformly distributed random numbers in *f*_*kl*_ ∈ [0, 1] and δ_*kl*_ ∈ [0, 2π] in the presence of temporal fluctuations (Figure 1D).

When the length of a bond becomes shorter than a threshold ϵ during the time evolution according to Equation (1), the bond rotates 90 degrees around its midpoint, and the five bonds of the two vertices at the rotated bond reconnect to achieve a T1 transition (Figure 1E).

The numerical simulation is performed based on Equations (1)–(5) by the simple Euler method with time discretization of the increment *dt* = 0.001. The T1 transition occurs when the length ℓ_*kl*_ of the bond 〈*kl*〉 becomes shorter than a given length ϵ = 0.005 (see also Figure 1F). The friction constant is set to be η_*i*_ = 1 except for the outer and inner walls. We choose the units of length and time so that the radius of outer wall and the coefficient for tension are given by *R*_out_ = 1 and γ_*c*_ = 1 (η_*i*_*R*_out_/γ_*c*_ = 1), respectively. The friction at the outer wall is given by η_*i*_ = 100, and at the inner wall as η_*i*_ = 10. The total number of cells is given by *N* = 450. Additional parameter values are given by *R*_in_ = 0.5, *k*_out_ = 100, *k*_in_ = 100, *K*_*a*_ = 10, $A_{0}=\left(\pi R_{\text{out}}^{2}-\pi R_{\text{in}}^{2}\right)/N\sim 0.0052$, and $L_{0}=\sqrt{4\pi A_{0}}\sim 0.26$. The strength of the tension *K*_*p*_ is shown in the figures. This set of parameter values are basically comparable with our previous work (Sato et al., 2015a) except for the total number *N* of cells, which was 168 in the previous work. By increase of *N*, we are able to investigate the mechanical processes during the collective cell movement by measuring spatiotemporal distributions and correlations of the velocity fluctuations of cell motility and the T1 transition frequency.

Note that the collective cell migration of genital disc of *Drosophila* is considered to be driven mainly by the activity at the apical side (Sato et al., 2015a). Therefore, in our model active processes were considered for the apical processes, such as the remodeling of cell-cell junctions. For basal processes, only a passive resistance force was considered between cells and the basal extracellular matrix and cells. A collective migration of epithelial cells may also be induced by protrusive activities along the basement membrane. Motivated by such basal processes, directional driving forces of individual cells have been introduced to a cell vertex model in self-propelled Voronoi model (Li and Sun, 2014; Garcia et al., 2015; Bi et al., 2016).

### Analysis of simulation results

In the following sections, from numerical simulations, we determine the cell angular velocity *v*, the elliptical cell shape anisotropy *s*, the frequency of T1 transition, *n*_T1_, and the bond chirality *c*. The angular velocity *v*_α_ of the α-th cell is given by *v*_α_ = *dψ*_α_(*t*)/*dt* around the origin (0,0) (genital disc center), where ψ_α_ is the angular position of the α-th cell around the origin, and the cell position is given by the centroid of the polygonal cell. The angular velocity *v*_α_ is positive when the cell moves in clock-wise direction. Then, *v* is given as the average of *v*_α_ among the cells. For the elliptical cell anisotropy *s*_α_ of the α-th cell, we first quantify the moment matrix of inertia **M** of the polygonal cell, and *s*_α_ is obtained by the half difference of the two eigenvalues of **M**. Thus, *s*_α_ measures the deviation of the cell shape from that of a circle. The variable *s* is given as the average of *s*_α_ among the cells. For the frequency of T1 transition, *n*_T1_, we count the number of T1 transition per unit time. For the bond chirality *c*, we count the number of tilted cell-cell junctions as described previously (Sato et al., 2015a). In this article, it is given by *c* ≡ *N*_cl_/*N*_ccl_ − *N*_ccl_/*N*_cl_, where *N*_cl_ and *N*_ccl_ are the numbers of cell-cell junctions tilted clockwise and counterclockwise, respectively, from the AP axis. When the distribution of the bond directions is uniform with respect to the AP axis, *c* is zero, while *c* is not zero when the bond directions are biased. The mathematical details of these quantities are given in the Appendix.

## Results

### Oscillation in collective cell movement

**Figure 2:**
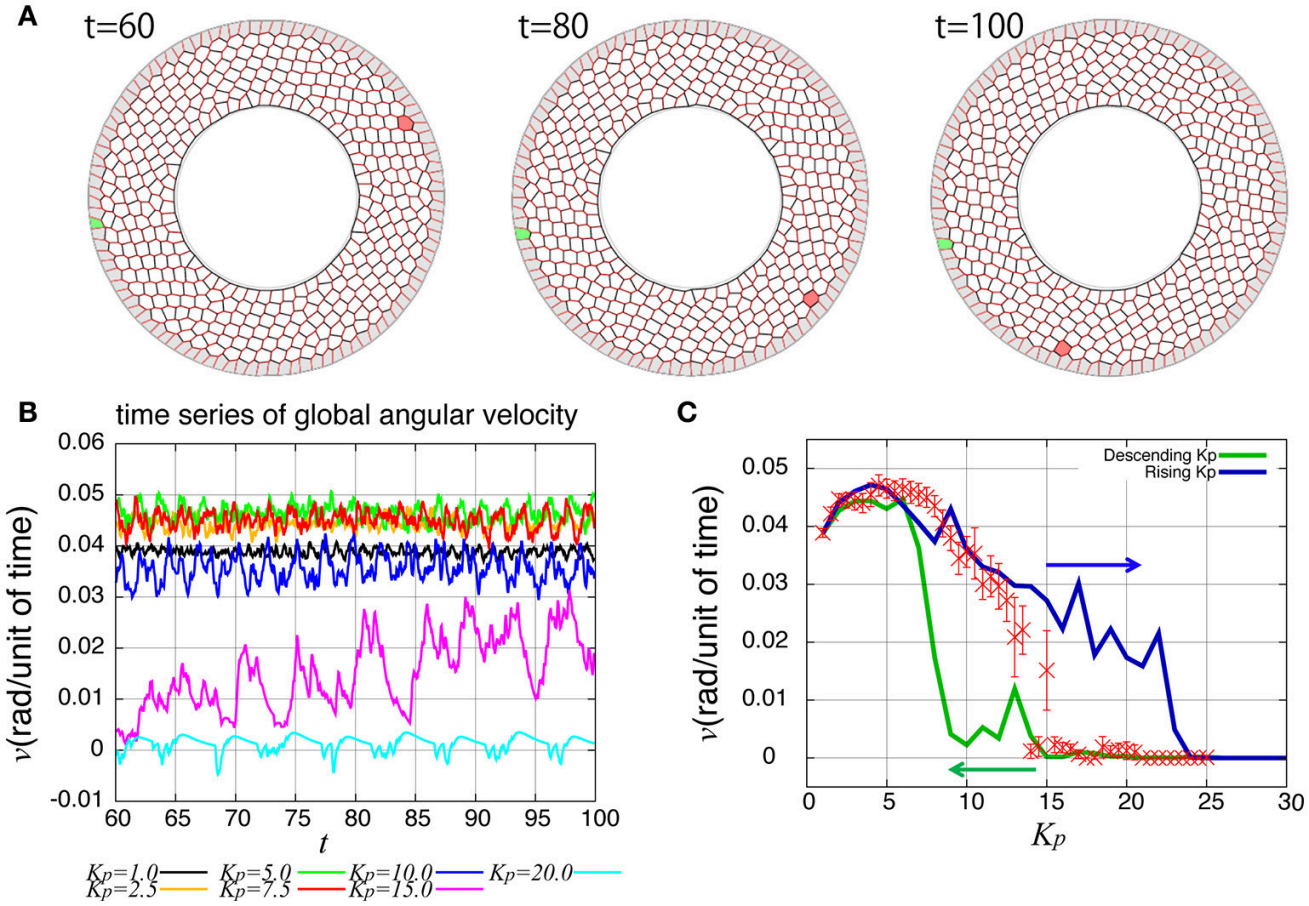
Rotational motion of the genital disc in the absence of line tension fluctuation. **(A)** Time-lapse images of genital disc rotation. **(B)** Time evolution of global angular velocity for various values of *K*_*p*_. **(C)** *K*_*p*_-dependence of the global angular velocity. The angular velocity was averaged over the time window from *t* = 60 to 100 (red). Error bars indicate the standard deviation in the time course. Hysteresis curves for the global angular velocity were plotted for increasing and decreasing parameter values (blue and green lines, respectively).

As reported previously, the entire ring-like epithelial tissue that surrounds the genitalia rotates in the clockwise direction (Figure 2A, Supplementary Movie 1). To see the dynamics of collective cell movement quantitatively, we first measured the global angular velocity for the case without temporal fluctuation in the tension, as plotted over time in Figure 2B. Here, the global angular velocity is the average of the angular velocity of all the cells. The global angular velocity exhibited a dependence on *K*_*p*_ as reported previously (Sato et al., 2015b). In Figure 2C (red symbols), the global angular velocity averaged over time was plotted against *K*_*p*_. The global angular velocity showed a maximum value at around *K*_*p*_ = 5. As *K*_*p*_ increased further, the state is discontinuously changed from the rotating state to a state without rotation at around *K*_*p*_ ≈ 15. Such an abrupt change is called subcritical transition. This subcritical characteristic of the transition was also seen in the hysteresis curves (blue and green lines).

**Figure 3:**
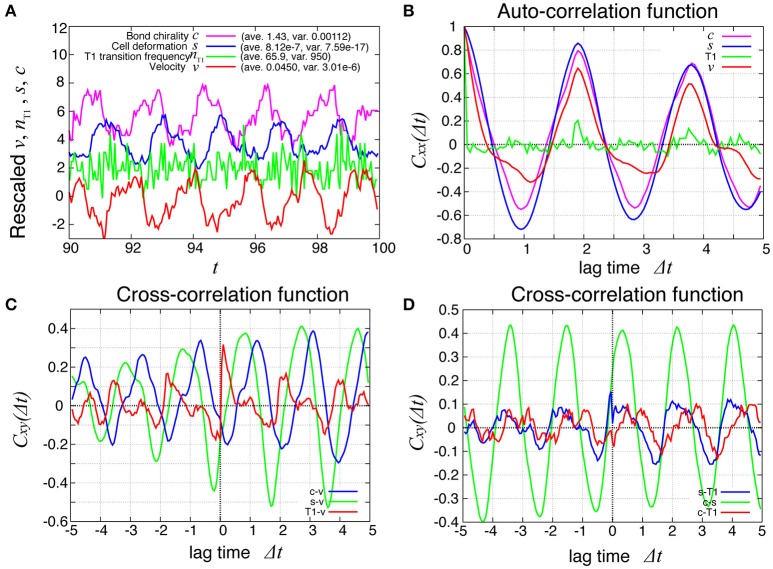
Dynamics and correlation functions for *K*_*p*_ = 7.5 in the absence of line tension oscillation, showing oscillations of the rotation velocity, T1 transition frequency, shape deformation and chirality in bond angles and correlations between them with time delays. **(A)** Time evolution of the global angular velocity *v*, (red), the number of T1 transitions per unit time, *n*_T1_ (green), elliptical anisotropy *s* (blue), and bond chirality *c* (purple). For these quantities of *x* = *v, n*_T1_, *s, or c*, we plotted the scaled values as (*x* − 〈*x*〉)/σ_*x*_ with the average 〈*x*〉 and standard deviation σ_*x*_. For visibility, the plots were offset by 2 (*n*_T1_), 4 (*s*), and 6 (*c*). **(B)** Auto-correlations of *v*, *n*_T1_, *s*, and *c*. **(C)** Cross-correlation functions between *v* and the other quantities, given by *C*_*xv*_(Δ*t*) = 〈δ*x*(*t*)δ*v*(*t* + Δ*t*)〉/σ_*x*_σ_*v*_, where *x* is *n*_T1_, *s*, or *c* (red, green, and blue, respectively). Here, the positive time difference Δ*t* means that *x* precedes *v*. δ*x* = *x* − 〈*x*〉. **(D)** Cross-correlation functions given by *C*_*sn*_T1__(Δ*t*) = 〈δ*s*(*t*)δ*n*_T1_(*t* + Δ*t*)〉/σ_*s*_σ_*n*_T1__, *C*_*cs*_(Δ*t*) = 〈δ*c*(*t*)δ*s*(*t* + Δ*t*)〉/σ_*c*_σ_*s*_, and *C*_*cn*_T1__(Δ*t*) = 〈δ*c*(*t*)δ*n*_T1_(*t* + Δ*t*)〉/σ_*c*_σ_*n*_T1__.

How are the cell motions organized during the collective movement? One possibility would be that the cells would move at the same speed. Another possibility would be that they move in the clockwise direction randomly without a cell-cell correlation. Interestingly, the global angular velocities exhibited temporal oscillatory behaviors as shown in Figure 2B, indicating that some temporal organization is present in the cell movements. To examine this oscillatory behavior more closely, in addition to the global angular velocity, we plotted the time series of the T1 transition frequency, *n*_T1_(*t*), the cell shape distortion *s*(*t*), and the bond chirality *c*(*t*) in Figure 3A (see Appendix for the detailed explanation of these quantities). To see the oscillatory behavior more statistically, the temporal auto-correlation functions were plotted in Figure 3B for the four quantities. The auto-correlation function quantifies the correlation of a quantity at different time points with lag time Δ*t*. This analysis indicated that these quantities exhibit oscillation with the same oscillation period of *t* ≈ 2. To determine the temporal order of the events, we next studied the temporal cross-correlation functions among the four quantities, shown in Figures 3C,D. The cross-correlation function quantifies the correlation of two quantities at different time points with lag time Δ*t*. This analysis revealed that as the bond chirality *c* increases, cell distortion is accumulated, as evidenced by the increase in *s* (green line in Figure 3D), accompanied by a decrease in the angular velocity, as indicated by the negative peak with a positive delay time in the blue line in Figure 3C. Such cell distortion can be released by T1 transition. Figure 3D (blue line) shows that the correlation between *n*_T1_ and *s* reaches its maximum positive value when the delay time is zero, indicating that an accumulation of distortion accompanies the increase in the number of T1 transition. Then, the T1 transition enhances the change in cell positions, causing an increase in the angular velocity *v*(*t*). The correlation between *v*(*t*) and *n*_T1_(*t*) reaches its maximum value at a small positive delay time (red line in Figure 3C).

### Spatial propagation of cell rearrangement

**Figure 4:**
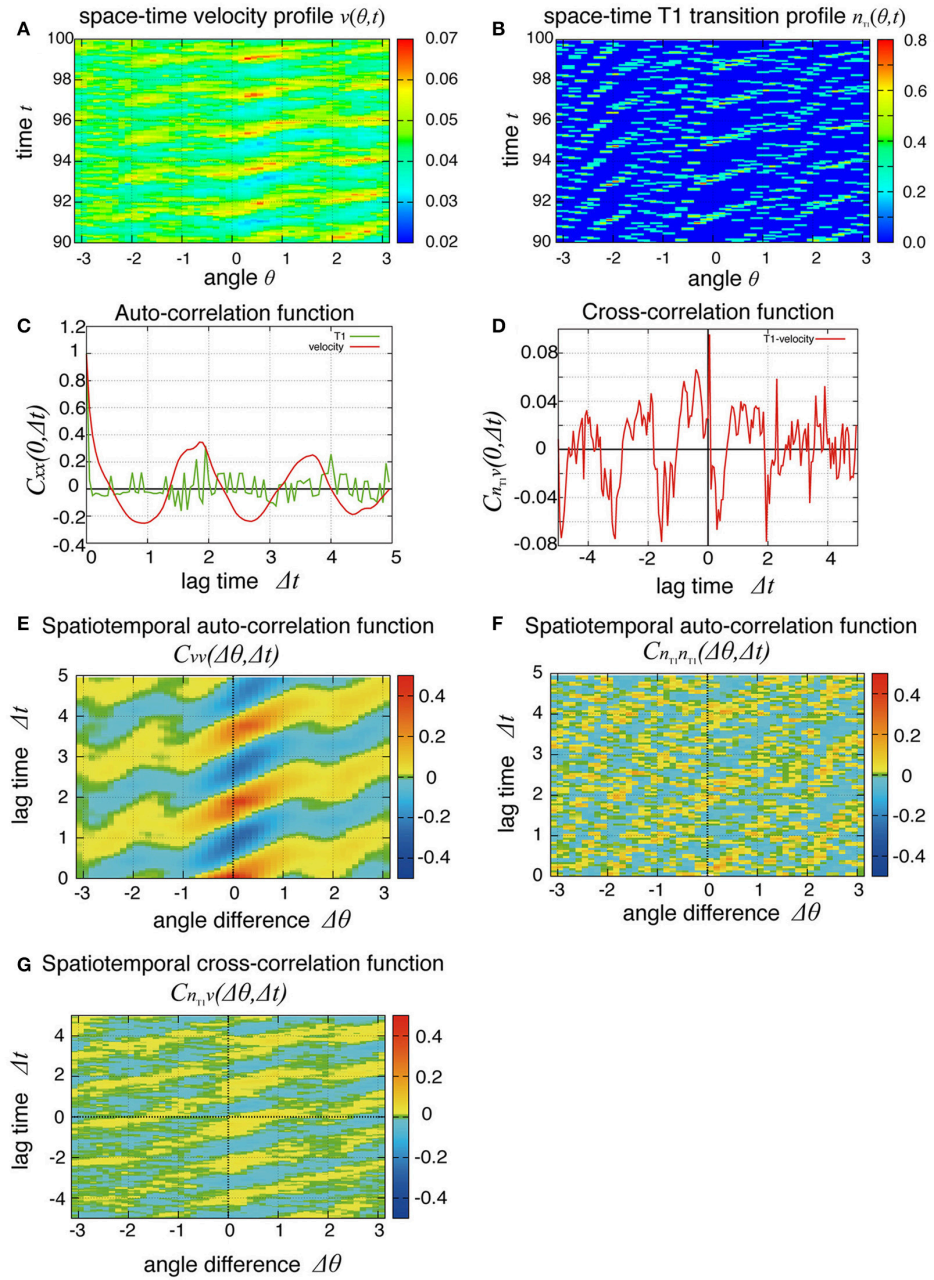
Spatial propagations of cellular motility and T1 transition frequency. **(A,B)** Spatiotemporal distributions of local angular velocities **(A)** and frequency of T1 transitions **(B)** for *K*_*p*_ = 7.5. **(C–G)** Spatiotemporal correlation functions for *K*_*p*_ = 7.5 in the absence of oscillation of chiral line tension. A schematic explanation of spatiotemporal correlation function is shown in Supplementary Figure 1. **(C)** Temporal auto-correlation of the local angular velocity (red line) and the T1 transition (green line). **(D)** Temporal cross-correlation function. **(E)** Spatiotemporal auto-correlation of the local angular velocity. **(F)** Spatiotemporal auto-correlation of the local frequency of T1 transition. The max/min color was plotted when the color value exceeded the max/min of the color bar. **(G)** Spatiotemporal cross-correlation *C*_*n*_T1_*v*_(Δθ, Δ*t*) = 〈δ*n*_T1_(θ, *t*) δ*v*(θ+Δ*θ, t*+Δ*t*)〉/σ_*n*_T1__σ_*v*_ between fluctuations of the local angular velocity and the frequency of T1 transition. θ and Δθ here are defined in the clockwise direction. **(F,G)** The auto-correlations **(F)** and the cross-correlations **(G)** along the time axis of Δθ = 0.

So far, we have studied the global quantities averaged over space. To see whether the cell movement is organized spatially, we next examined the cell movement and rearrangements in a local area. In Figure 4, the local averages of the angular velocities and the T1 transition frequency, *v*(θ, *t*) and *n*_T1_(θ, *t*) are shown in Figures 4A,B, respectively (the last 10 units of time are displayed). We found small patches indicating propagations of increase in both angular velocity and T1 transition frequency generated at different positions. We then scrutinized these propagation patterns in more quantitative ways as follows. We first considered the temporal autocorrelations of the local angular velocity *v* (Figure 4C, red line), which also exhibited an oscillatory behavior with period of about 2 units of time, consistent with the case of global velocity (Figure 3B). Then, we calculated the spatiotemporal autocorrelation function of the local angular velocity as shown in Figure 4E (a schematic explanation of spatiotemporal correlation function is shown in Supplementary Figure 1). The peak in the correlation at *t* = 0 and Δθ = 0 exhibited a propagation in the clockwise direction Δθ > 0 with a velocity of about 2 (rad) per unit time. Interestingly, this propagation speed was much faster than the angular velocity of cell movement, which was at most about 0.05 (rad) per unit time (Figure 2C, *K*_*p*_ = 5). Considering that in the present model the potential force is balanced with the frictional force that is proportional to the velocity, the propagation of velocity change indicates that the force is transmitted through the tissue as in Introduction.

To see the propagation of the T1 transition statistically, we next plotted the temporal and spatiotemporal auto-correlation functions in Figure 4C (green line) and Figure 4F, respectively. When compared with the local velocity [Figure 4C (red line) and Figure 4E], the oscillation and propagation of the T1 transitions were not so evident. Then, the cross-correlation function clearly indicated a correlation between the local quantities (Figures 4D,G), indicating the oscillation and propagation behaviors of the T1 transition. The temporal cross-correlation function reached a maximum value with a small positive lag time (Figure 4D), and the correlation propagated spatially in the clockwise direction (Figure 4G). This analysis of cross-correlation functions indicated that after the propagation of the T1 transition passes through, the propagation of the increase in angular velocity occurs.

**Figure 5:**
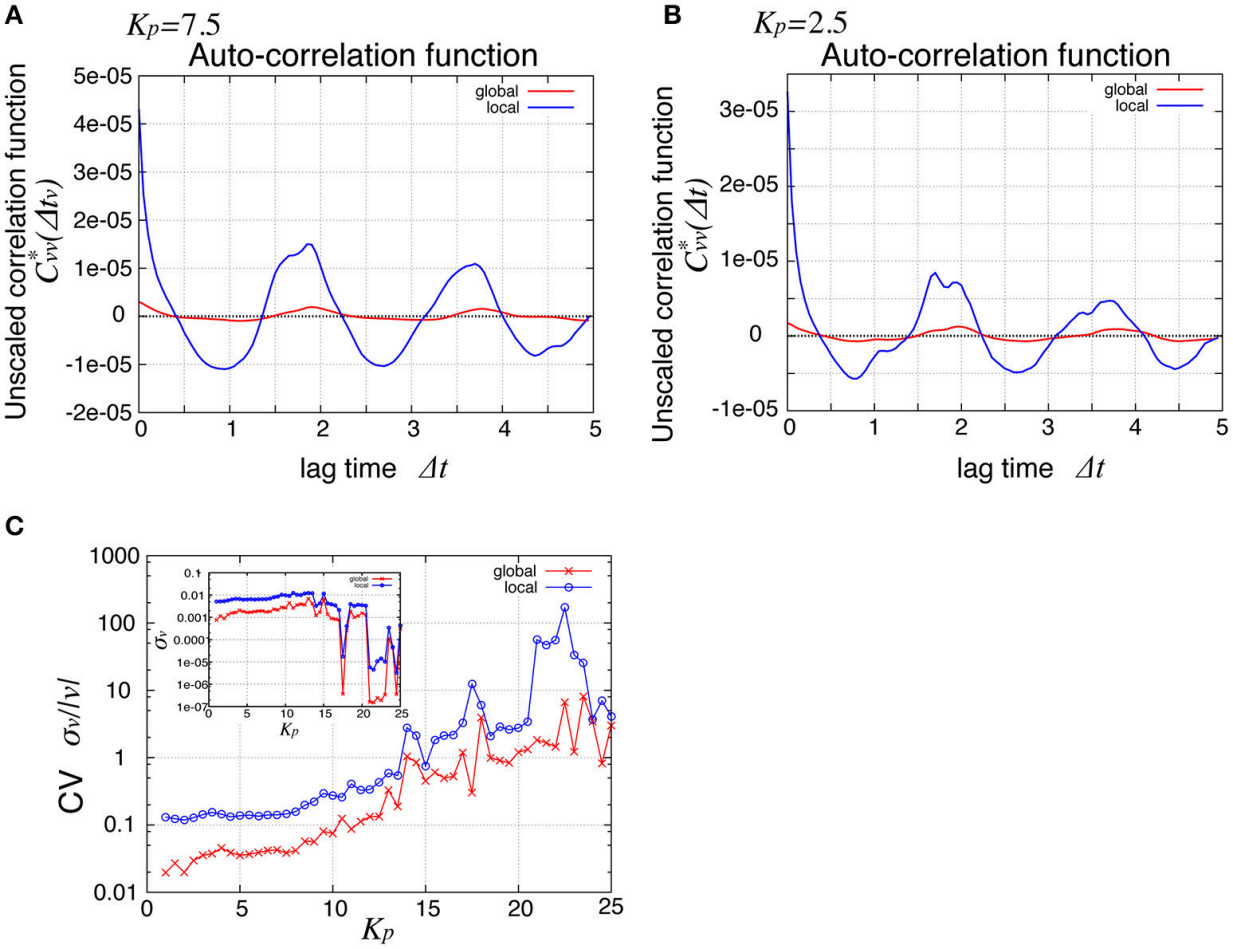
Comparison of global and local behaviors, suggesting the relationship between the spatial propagation and the global oscillation of cell rearrangements. **(A,B)** Auto-correlation functions $C_{vv}^{*}\left(\Delta t\right)=\langle \delta v\left(t\right)\delta v\left(t+\Delta t\right)\rangle$ of the angular velocities for *K*_*p*_ = 7.5 **(A)** and *K*_*p*_ = 2.5 **(B)**. **(C)** Standard deviation scaled by the average time courses of the angular velocity. Red symbols, for global velocity; blue symbols, local velocity. Inset shows their standard deviation.

To see the mechanism of the global oscillation in Figure 3, we next considered the relationship between this spatial propagation of cell rearrangement and the global oscillation. For both the global and local angular velocities, the auto-correlation functions without normalization by their variances were plotted in Figures 5A,B for *K*_*p*_ = 2.5 and 7.5, respectively. We found that the peak correlations at Δ*t* ~ 1.9 and 3.7 as well as the variances (Δ*t* = 0) of the local velocity (blue lines) were about 10 times larger than those of the global velocity (red line). We then examined the *K*_*p*_-dependence of the global and local velocities. In Figure 5C, for various *K*_*p*_ values, we plotted the coefficient of variation σ_*v*_/〈*v*〉 (CV) for the global and local velocities as red crosses and blue circles, respectively. Here, σ and 〈*v*〉 were the standard deviation and the temporal average of velocity, respectively. As shown in Figure 2C, the global angular velocity decreased as *K*_*p*_ decreased below *K*_*p*_ = 5. The CV for the local velocity was about ten times larger than that for the global velocity. These differences in both the auto-correlation functions and CV could stem from the propagation of cell rearrangement. Changes in the local velocity showed temporal oscillatory fluctuations, which propagated spatially. Because of this propagation, the phase of oscillation in the velocity was distributed through the entire system. Therefore, the amplitude of oscillation in the global velocity was averaged out by the summation of local velocities in the entire system. The proportion of the different phases of oscillation in the entire system was time-dependent. Such time-dependence might arise due to the fact that the system size is incommensurate with the wavelength. For a sufficiently large system, the oscillation in the global velocity may become negligible. Such inconsistency was clearly seen in the spatiotemporal profile of the local velocity shown in Figure 4A, where continuous propagations were interrupted by a sudden change in the phase of oscillation.

### Propagation of cell rearrangement depends on the chirality

**Figure 6:**
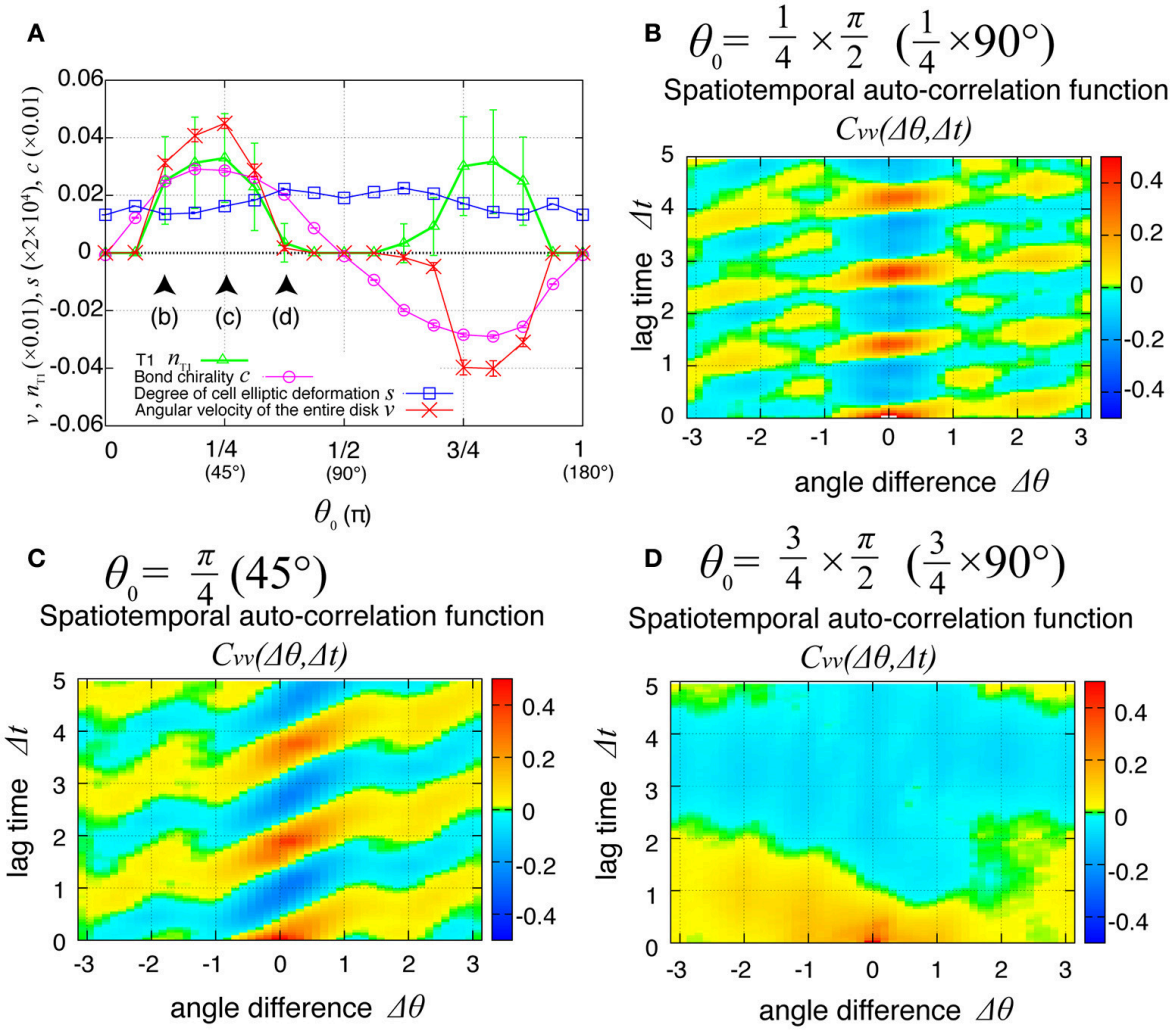
Dependence of collective cell movement on the chirality in the absence of tension fluctuation. **(A)** Dependence of average quantities on the angle θ_0_ in Equation (6): *v* (red), *n*_T1_ (green), *s* (blue), and *c* (purple). To calculate the *K*_*p*_-dependence of the angular velocity, the time window from *t* = 60 to 100 was used for this average. Vertical bars indicate standard deviation in the time course. **(B–D)** Dependency of spatiotemporal correlation functions of fluctuations in locally averaged cell angular velocity $C_{vv}\left(\Delta \theta ,\Delta t\right)=\langle \delta v\left(\theta ,t\right)\delta v\left(\theta +\Delta \theta ,t+\Delta t\right)\rangle /\sigma_{v}^{2}$ for *K*_*p*_ = 7.5 in the absence of oscillation of chiral line tension; **(B)** for $\theta_{0}=22.5^{{}^{\circ}}$, **(C)** for $\theta_{0}=45^{{}^{\circ}}$, and **(D)** for $\theta_{0}=67.5^{{}^{\circ}}$.

The direction of the collective cell movement depends on the chiral property θ_0_ in tension in Equation (6). Figure 6A shows that the angular velocity *v* and the bond chirality *c* exhibited their maximum and minimum values at $\theta_{0}=45^{{}^{\circ}}$ and $\theta_{0}\;=\;135^{{}^{\circ}}$, respectively, and that the T1 transitions occurred most frequently at both these angles, as reported previously (Sato et al., 2015b). The bond chirality *c* disappearred at $\theta_{0}=0,90,180^{{}^{\circ}}$, as did the angular velocity and T1 transition. In Figures 6B–D, the spatiotemporal correlations of the local angular velocities were plotted. We found that the propagation speed of the spatiotemporal correlation in local velocity fluctuations depended on the chiral property θ_0_ in tension.

### The cell rearrangement propagates through the system with tension fluctuations

**Figure 7:**
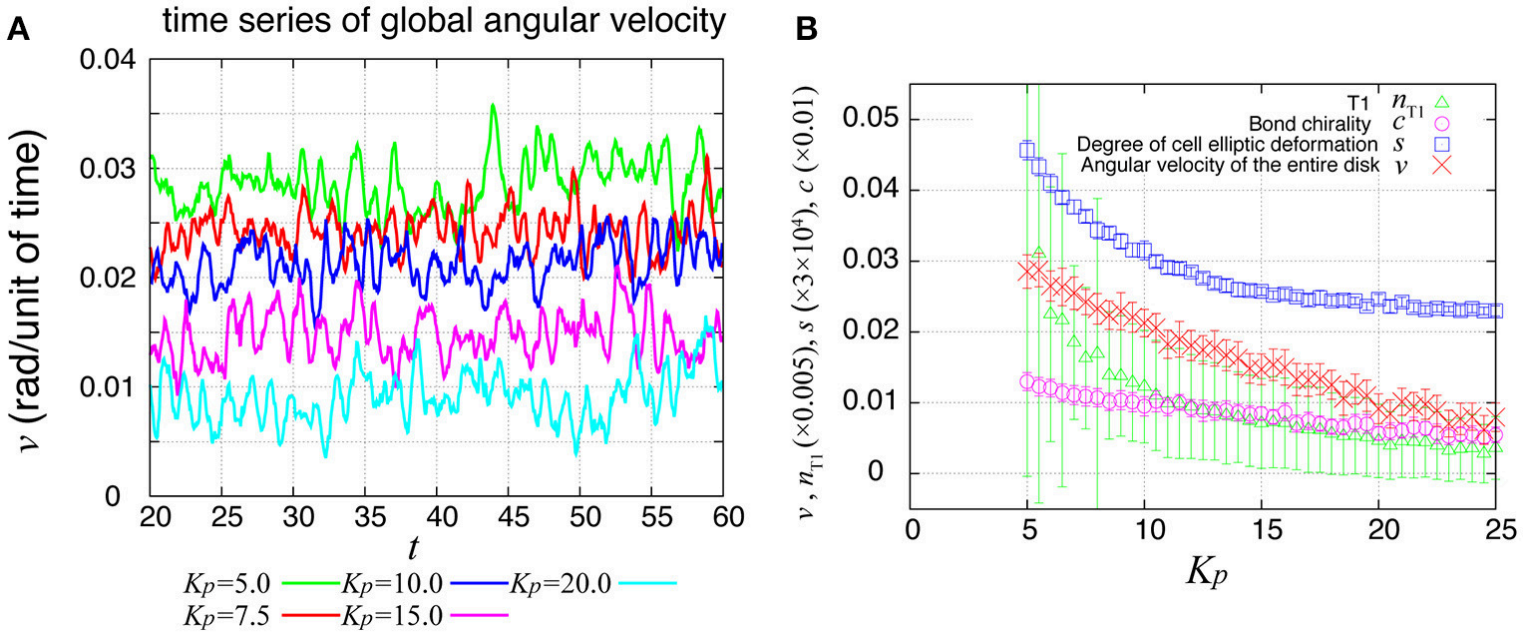
Global angular velocity in the presence of line tension fluctuation. **(A)** Time evolution of the global angular velocity of an entire ring-like tissue for various values of *K*_*p*_. **(B)** *K*_*p*_-dependence of the global angular velocity. To calculate the *K*_*p*_-dependence, the angular velocity was averaged over the time window from *t* = 20 to 60. Error bars indicate standard deviation in the time course.

During the rotation of *Drosophila* genital disc, the contraction of the cell-cell junctions has been shown to be accompanied by temporal fluctuations (Sato et al., 2015a). The myosin II intensity at the junctions has also exhibited temporal fluctuations in an inversely correlated way. Therefore, we next asked whether the same propagation of velocity change and T1 transitions occur in the collective cell movement of epithelial cells under this condition. To consider the fluctuating contraction, we introduced a temporal fluctuation in the tension γ_*kl*_ as shown in Equation (7). The numerical simulation of the case with tension fluctuation is shown in Supplementary Movie 2. We found that an oscillation of the global angular velocities also occurred in this condition with tension fluctuation (Figure 7A), although the oscillation was more irregular than in the case without tension fluctuation (Figure 2B). As *K*_*p*_ increased, the global angular velocity continuously decreased without an apparent transition (Figure 7B, in contrast to the previous case without fluctuations in Figure 2C).

**Figure 8:**
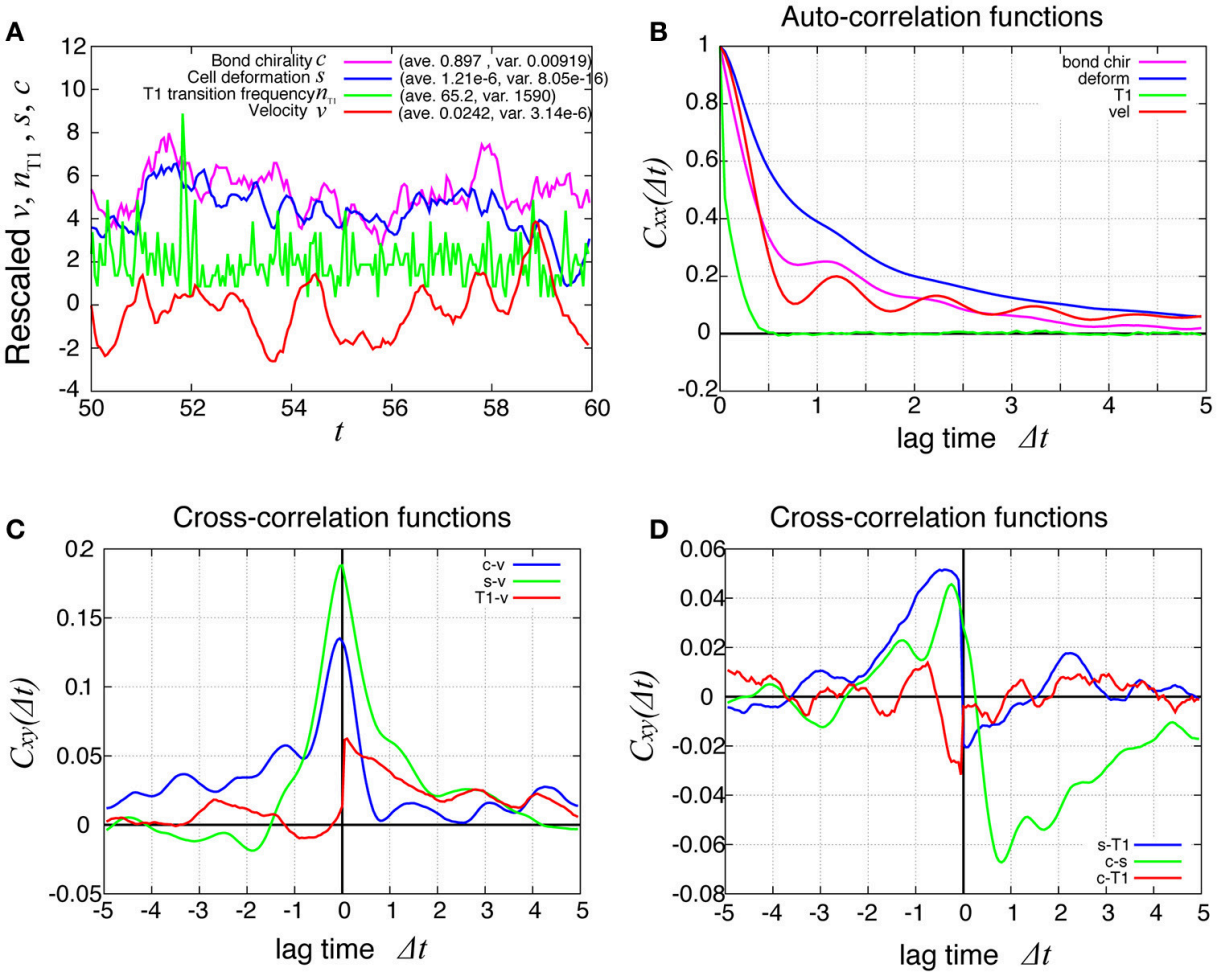
Dynamics and correlation functions for *K*_*p*_ = 7.5 in the presence of line tension fluctuation, showing a weak oscillation in cell motility and the correlations with other quantities. **(A)** Time evolutions of the average angular velocity (red curve), the number of T1 transitions per unit time (green curve), the average *s* (blue curve), and the bond chirality *c* (purple curve) in the time window from *t* = 50 to 60. The quantities *x* = *v, n*_T1_, *s, c* plotted in this figure were rescaled as (*x* − 〈*x*〉)/σ_*x*_, respectively, with the average 〈*x*〉 and standard deviation σ_*x*_. The plots are offset by 2 (*n*_T1_), 4 (*s*), and 6 (*c*), for visibility. **(B)** Auto-correlations of *v, n*_T1_, *s*, and *c*. **(C)** Cross-correlation functions between *v* and the other quantities, given by *C*_*xv*_(Δ*t*) = 〈δ*x*(*t*)δ*v*(*t* + Δ*t*)〉/σ_*x*_σ_*v*_, where *x* is *n*_T1_, *s*, or *c* (red, green, and blue, respectively). Here, the positive time difference Δ*t* means that *x* precedes *v*. δ*x* = *x* − 〈*x*〉. **(D)** Cross-correlation functions given by *C*_*sn*_T1__(Δ*t*) = 〈δ*s*(*t*)δ*n*_T1_(*t* + Δ*t*)〉/σ_*s*_σ_*n*_T1__, *C*_*cs*_(Δ*t*) = 〈δ*c*(*t*)δ*s*(*t* + Δ*t*)〉/σ_*c*_σ_*s*_, and *C*_*cn*_T1__(Δ*t*) = 〈δ*c*(*t*)δ*n*_T1_(*t* + Δ*t*)〉/σ_*c*_σ_*n*_T1__.

In Figure 8A (red line), the temporal evolution of the global angular velocity was plotted. As evident from the auto-correlation functions in Figure 8B, the rotating motion was more irregular compared to the case without tension fluctuation (Figure 3). The temporal cross-correlation between *v* and *n*_T1_ (red line in Figure 8C) indicated that the angular velocity increased or decreased suddenly after the frequency of T1 transition increased or decreased, respectively. The cross-correlation between *s* and *n*_T1_ reached its positive maximum value with a negative delay time (blue line in Figure 8D), suggesting that the cell distortion increased or decreased after the frequency of T1 transition increased or decreased, respectively.

**Figure 9:**
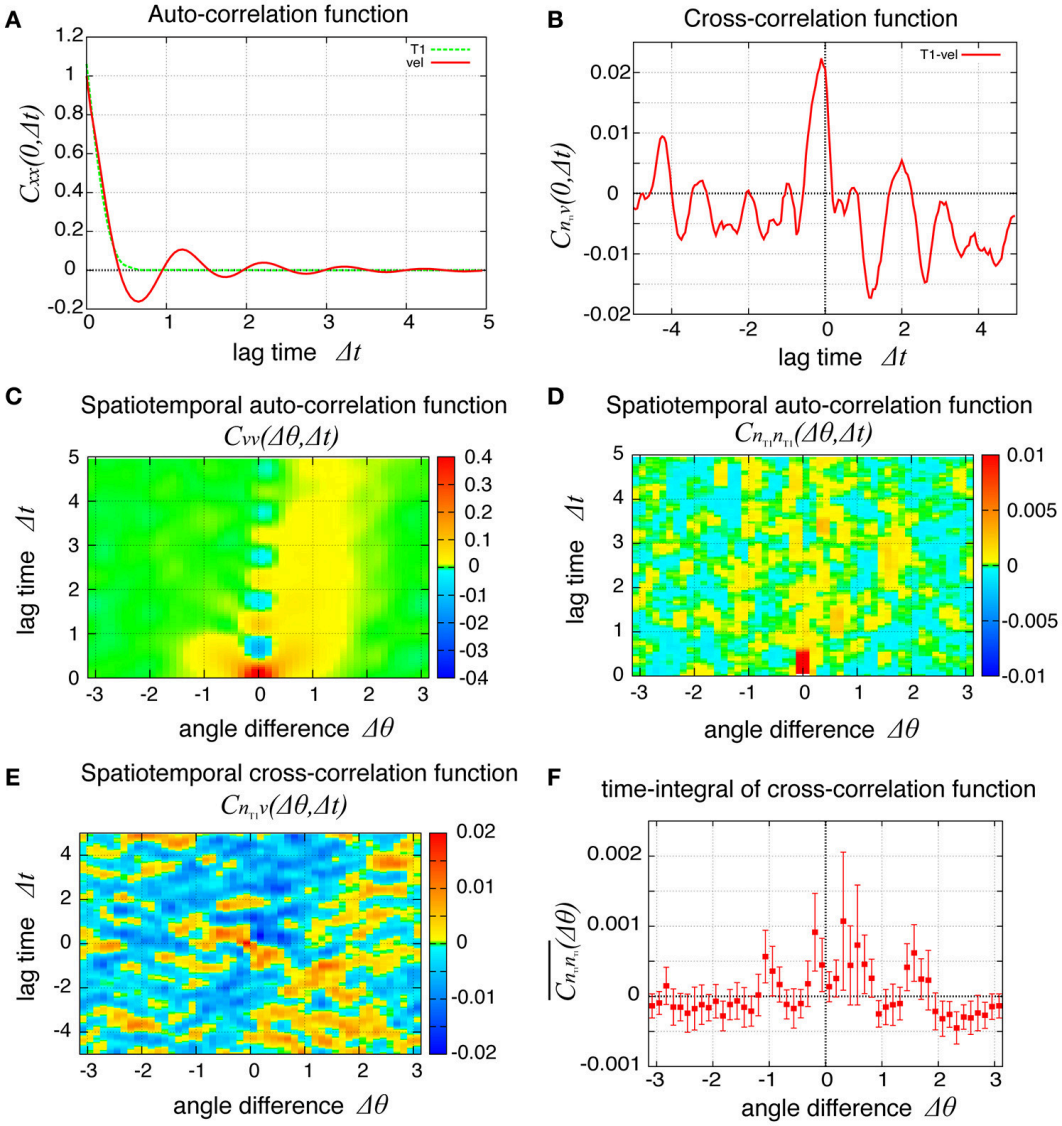
Spatiotemporal correlation functions for *K*_*p*_ = 7.5 in the presence of chiral line tension fluctuation, showing the spatial propagation of cell rearrangements. **(A)** Temporal auto-correlation function of the local angular velocity and the local frequency of T1 transitions (red and green lines, respectively). **(B)** Temporal cross-correlation function between the two quantities. **(C,D)** Spatiotemporal correlation function of the local angular velocity **(C)** and the local frequency of T1 transition **(D)**. **(E)** Spatiotemporal correlation function between the two quantities. **(F)** Integrated spatiotemporal correlation $\bar{C_{n_{\text{T}1}n_{\text{T}1}}}\left(\Delta \theta \right)$ of **(D)** along the time difference between Δ*t* = 1 and Δ*t* = 5, given by $\bar{C_{n_{\text{T}1}n_{\text{T}1}}}\left(\Delta \theta \right)\;=\overset{5}{\underset{1}{\int }}C_{n_{\text{T}1}n_{\text{T}1}}\left(\Delta \theta ,\Delta t\right)d\left(\Delta t\right)$, where *C* is the spatiotemporal correlation function. To obtained the plot, 132 simulations were carried out with different values of the random parameters, the frequency *f*_*kl*_ and the initial phase δ_*kl*_ of tension fluctuation. The integration $\bar{C_{n_{\text{T}1}n_{\text{T}1}}}\left(\Delta \theta \right)$ was first performed for each simulation. Then, the means over the 132 simulations were plotted (marks) against the angle difference Δθ. Vertical bars indicate 95 percent confidence intervals of the means among the 132 simulations.

To examine the propagation of velocity fluctuation, the temporal auto-correlation functions of the local angular velocity *v* was depicted in Figure 9A; it quickly decayed with oscillation with a period of about 1.2, which agreed with the small peak in the auto-correlation function of the global angular velocity in Figure 9B (red line). In Figure 9C, although the peak at the origin also decayed quickly in space, it exhibited a propagation in both clockwise and counter-clockwise directions, in contrast to the previous case without tension fluctuation. The propagation in the counter-clockwise direction ceased in about one unit of time and 1 rad. In contrast, the propagation in the clockwise direction, seen as the yellow region, continued for more than five units of time (Δ*t* = 5). The propagation speed was much slower than in the previous case without tension fluctuation. The spatiotemporal auto-correlation function of the local frequency of T1 transition did not indicate a clear propagation behavior (Figure 9D). However, the integral of the correlation function with respect to time showed that the correlated behavior was seen more in the clockwise direction than the other (Figure 9F). The cross-correlation between the angular velocity and the T1 transition was plotted (Figure 9E), and showed that the correlated behavior indicated by yellow was more evident in the clockwise direction. Although the propagation speed was much slower than in the case without tension fluctuation, it was still much faster than the angular velocity of cell movement (about 0.025 rad per unit time for *K*_*p*_ = 7.5). We conclude that the propagation of the cell rearrangement is a robust property of the epithelial cells' collective cell movement.

**Figure 10:**
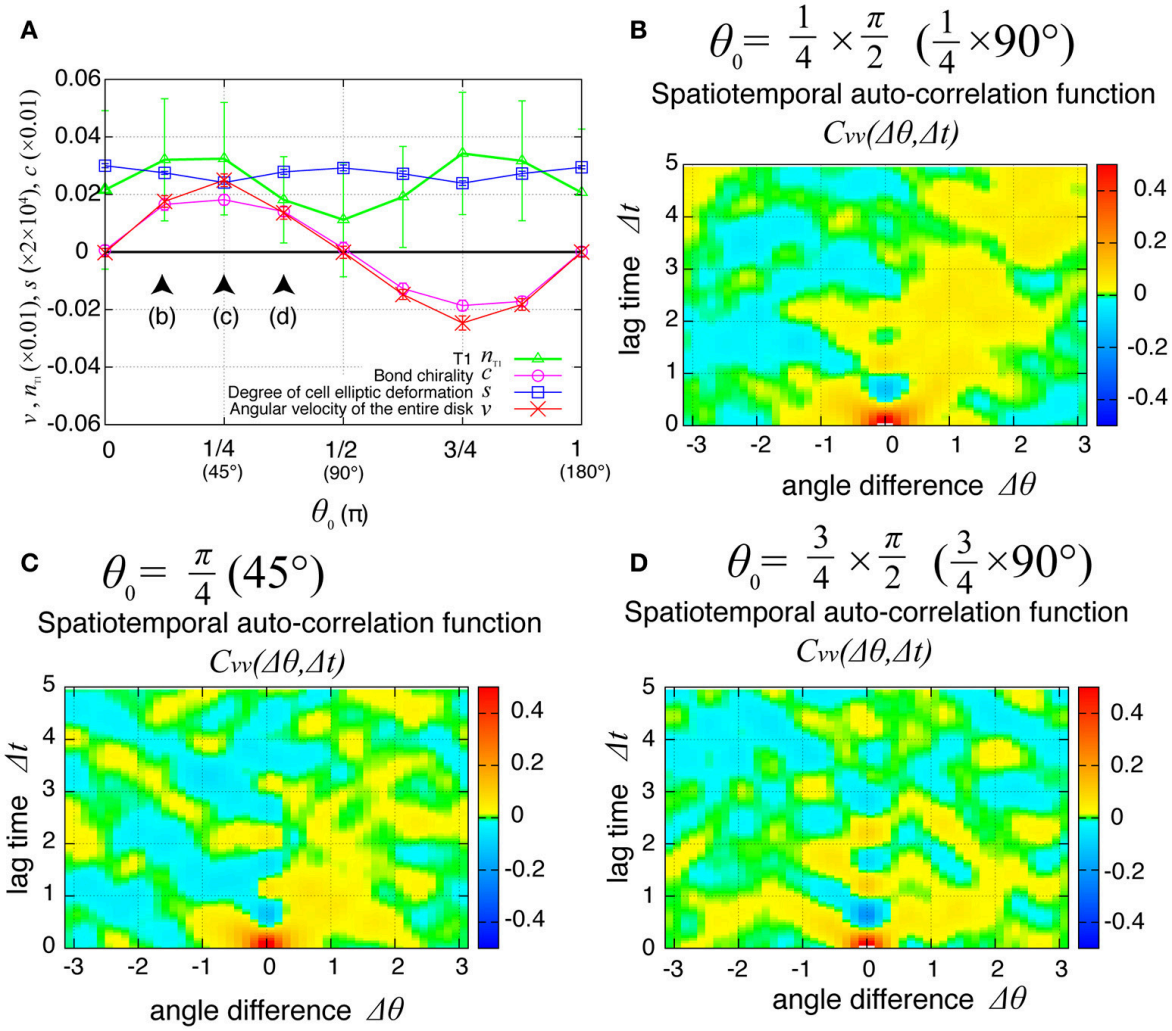
Dependence of collective cell movement on chirality in the presence of tension fluctuation. **(A)** Dependencies of average quantities on the angle θ_0_ in Equation (6). **(B–D)** Dependence of spatiotemporal correlation functions of fluctuations in the local angular velocity $C_{vv}\left(\Delta \theta ,\Delta t\right)=\langle \delta v\left(\theta ,t\right)\delta v\left(\theta +\Delta \theta ,t+\Delta t\right)\rangle /\sigma_{v}^{2}$ for *K*_*p*_ = 7.5, **(B)** for $\theta_{0}=22.5^{{}^{\circ}}$, **(C)** for $\theta_{0}=45^{{}^{\circ}}$, and **(D)** for $\theta_{0}=67.5^{{}^{\circ}}$. See Figure 6 for details.

Finally, we examined the dependence of the collective cell movement on the chiral property θ_0_ in tension in Equation (6) in the presence of tension fluctuation. As shown in Figure 10A, the angular velocities *v*, the bond chirality *c*, the T1 transition *n*_T1_, and the cell shape deformation *s* exhibited smooth dependences on the chiral direction θ_0_ with the same tendency as in the case without tension fluctuation shown in Figure 6A. In Figures 10B–D, the spatiotemporal correlation of the local angular velocities was plotted for different values of θ_0_, and showed that the propagation of the fluctuation in the local velocity fluctuations occurred in the clockwise direction for these cases.

## Discussion

In this study, we investigated the spatiotemporal self-organization of junctional remodeling and cell rearrangement in the collective cell movement of epithelial cells based on a numerical cellular vertex model. For this analysis, we focused on the rotating motion of a ring-like tissue composed of epithelial cells that exhibit a chiral contraction of cell-cell junctions. In the absence of spatiotemporal patterns in molecular signaling activity, the spatiotemporal organization of T1 transition occurred uninterruptedly in time, so that the tissue rotated in a specific direction, as we reported previously (Sato et al., 2015a). We further found in this study that the sequence of T1 transitions occurred as a traveling wave in the following way. When a cell changed its position owing to contraction and T1 transitions, it increased stress at the surrounding cells, leading to an increase in the frequency of T1 transition at the neighboring cells. Chiral contraction could bias the propagation direction by modulating this frequency in a direction-dependent manner. Consequently, the cell rearrangements propagated in a specific direction as a traveling wave, which drove the rotation of the epithelial ring-like tissue. This traveling wave behavior occurred in both the absence and presence of tension fluctuation.

**Figure 11:**
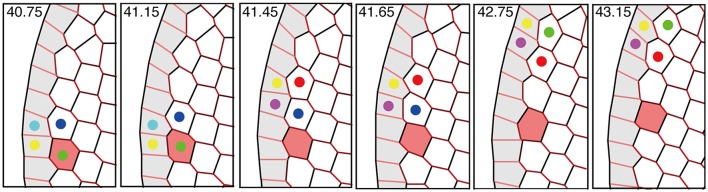
Propagation of T1 transition and cell rearrangements. T1 transition occurs between cells (indicated by colored dots) that undergo cell intercalation. The propagation of T1 transition is much faster than the cell movement (see red-colored cell). This figure was obtained from our numerical simulation in the absence of tension fluctuations (*Kp* = 7.5).

Interestingly, the speed of the traveling wave of T1 transition and cell rearrangement in this mechanism was found to be much faster than the motile speed of individual cells, as shown in Figure 11 and Supplementary Movie 1. This finding indicates that in some developmental processes, morphogenetic information may be transmitted much faster and farther through the mechanical coupling reported in this article than through cell movement alone.

Our simulation result naturally arises a question as to whether the similar traveling wave behavior can be observed experimentally, in particular, the genitalia rotation in *Drosophila* male terminalia (Sato et al., 2015a). In the presence of tension fluctuation, the propagation of velocity change is difficult to be seen in a single time course as shown in Supplementary Movie 2, contrasting to the case without tension fluctuation as shown in Supplementary Movie 1. To see the traveling wave behaviors in the spatiotemporal correlation function, we had to take statistics for sufficiently enough number of samples. This is also the case for experimental situations, where fluctuations are inevitably present. To confirm whether the traveling wave behavior occurs in the tissue, a statistical analysis for several samples with image segmentation needs to be carried out, which remains to be a future topic for the study of tissue morphogenesis dynamics.

Another mechanism of collective epithelial cell movement is observed in *Drosophila* follicular epithelium (Haigo and Bilder, 2011; Cetera et al., 2014; Chen et al., 2016; Barlan et al., 2017). Elongating follicles are known to rotate circumferentially around their long axes driven by the epithelial cell migration (Haigo and Bilder, 2011). For this, the cells use the mechanism taking advantage of basal protrusion activities, which is explained in the Model and Method section. The same mechanism of collective epithelial cell movement is reported for the *in-vitro* monolayer sheet of MDCK cells (Serra-Picamal et al., 2012). Interestingly, a mechanical wave propagation, such as propagations of cellular velocities, rate of cell deformation and monolayer stresses, has been observed also in such a system (Serra-Picamal et al., 2012). In contrast to these examples, the *Drosophila* genitalia rotation relies not on the basal protrusive activity but only on the contraction activity of apical junctions (Sato et al., 2015a), as considered in the present paper. Whether a further generic principle for collective cell migrations underlies both apical and basal mechanisms is an intriguing future topic in both studies of biophysics and tissue morphogenesis.

Our model predicts that a non-uniform spatiotemporal patterning of the motile behaviors of cells including locomotion and T1 transition can be observed in the epithelial dynamics. We found that, accompanied by the transmission of an increase or decrease in the frequency of T1 transition (Figures 4F, 9D), the transmission of an increase or decrease in the local locomotion velocity of cells was observed (Figures 4E, 9C). Here we refer to the transmission of an increase or decrease in local locomotion velocity as “motility transmission.” This phenomenon may have analogies in the motion of particles, granules, or other elements in a crowded situation. A typical example is a traffic jam, where the congestion at the rear end spreads in time and space, which has been extensively studied in the field of physics (Sugiyama et al., 2008). Collective cellular motions in crowded situations and their spatiotemporal motility patterns have also been studied for human bronchial epithelial cell (HBEC) monolayers and numerical models of epithelial cells spontaneously migrating on a substrate (Garcia et al., 2015; Bi et al., 2016). A challenging future problem will be to reveal the general rules that determine the directionality and speed of motility transmissions, by comparing the dynamics of transmission and the spreading of motility in various crowded situations. The biological functions and possible applications of this phenomenon are also important future topics.

## Author contributions

TH and TS conceived the project and designed the numerical simulations. TH performed the numerical simulations. TH, EK, and TS wrote the manuscript.

### Conflict of interest statement

The authors declare that the research was conducted in the absence of any commercial or financial relationships that could be construed as a potential conflict of interest.

## Supplementary material

Supplementary Figure 1 Schematic explanation of spatiotemporal correlation function.

Supplementary Movie 1 Numerical simulation of the vertex model without tension fluctuations corresponding to Figure 3.

Supplementary Movie 2 Numerical simulation of the vertex model with tension fluctuations corresponding to Figure 8.

## Appendix

We calculated various quantities for our analyses as follows.

*Average cell angular velocity v*(*t*) = 〈*d*_ψ_α__/*dt*〉_global_ *among the entire tissue*. Cell angular velocity is defined in this article as the increase in the angle of the α-th cell location in the clock-wise direction in unit time *dψ*_α_/*dt* around the center of the ring-like tissue. ψ_α_ is given by the angle between an arbitrary direction and the direction of the center of the cell seen from the center of the disc, *i.e*., through the relationships $\text{cos}\left(-\psi_{\alpha }\right)=X/\sqrt{X^{2}+Y^{2}}$ and $\text{sin}\left(-\psi_{\alpha }\right)=Y/\sqrt{X^{2}+Y^{2}}$ with the position vector (*X, Y*) of the center of mass of the cell from the origin (0, 0) at the disc center. We calculate the center of mass of the cell consisting of vertices *i* = 1, 2, ⋯ , *I* designated in the counter-clockwise direction by

(A1) $$\begin{array}{l} X=\frac{1}{6A}\overset{I}{\underset{i=1}{\sum }}\left({\text{r}}_{i}\times {\text{r}}_{i+1}\right)\left(x_{i}+x_{i+1}\right), \end{array}$$

and

(A2) $$\begin{array}{l} Y=\frac{1}{6A}\overset{I}{\underset{i=1}{\sum }}\left(-{\text{r}}_{i}\times {\text{r}}_{i+1}\right)\left(y_{i}+y_{i+1}\right), \end{array}$$

with the position vector **r**_*i*_ = (*x*_*i*_, *y*_*i*_) of the *i*-th vertex from an arbitrary position and the cell area *A*. The cell area is calculated by

(A3) $$\begin{array}{l} A=\frac{1}{2}\overset{I}{\underset{i=1}{\sum }}\left({\text{r}}_{i}\times {\text{r}}_{i+1}\right). \end{array}$$

The *i* = (*I* + 1)-th vertex is identified with the *i* = 1-th vertex. The symbol × indicates a cross product in two dimensions, i.e., (*a*_1_, *a*_2_) × (*b*_1_, *b*_2_) = *a*_1_*b*_2_ − *b*_1_*a*_2_. The average 〈·〉 is performed among all the cells.

*Local average* θ(*t*) = 〈*d*_ψ_α__/*dt*〉_local(θ)_ *of the cell angular velocity*. We assign the angle θ of an arbitrary position (*x, y*) into *J* grids of the width 2π/*J*, as 2π(*j* − 1/2)/*J* ≤ θ < 2π(*j* + 1/2)/*J* with *j* = 1, 2, ⋯ , *J*. The grid number is set at *J* = 50. The angle θ is defined by $\text{cos}\left(-\theta \right)\;=x/\sqrt{x^{2}+y^{2}}$ and $\text{sin}\left(-\theta \right)\;=y/\sqrt{x^{2}+y^{2}}$. Furthermore, we take the average 〈·〉_Local_ of the angular velocity among the cells whose center of mass (*X, Y*) is located within each grid, 2π(*j* − 1/2)/*J* ≤ ψ_α_(*X, Y*) < 2π(*j* + 1/2)/*J*, at a later time step to define the velocity. Here, the angle ψ_α_ of the α-th cell location direction is defined as above.

*Total number n*_T1_(*t*) *of T1 transition events that occur between two adjacent time steps*. We define *n*_T1_(*t*) by counting the total number of T1 transitions that occur between two adjacent time steps and dividing it by the step size *dt* of time.

*Number density n*_T1_(θ, *t*) *of T1 transitions at time t*. We assign the spatial angle into grids as described above and count the number of T1 transitions that occur within each grid, 2π(*j* − 1/2)/*J* ≤ ϕ < 2π(*j* + 1/2)/*J* in the time interval from *t* to *t* + *dt*. The grid number is set as *J* = 50 as above. The angle ϕ of the bond location is defined by $\text{cos}\left(-\phi \right)\;=X/\sqrt{X^{2}+Y^{2}}$ and $\text{sin}\left(-\phi \right)\;=Y/\sqrt{X^{2}+Y^{2}}$ of the midpoint (*X, Y*) of the bond being examined. The number density *n*_T1_(θ, *t*) is given by dividing the number of T1 transitions by the time step size *dt*.

*Elliptical anisotropy s*(*t*) *in the shape of each cell*. We define the amplitude *s* of the elliptical deformation in the following way. First, we calculate the moment of inertia *M* for a uniformly thin plate, the mass density of which per area is scaled to be 1, taking the same shape as the cell being examined (Aigouy et al., 2010).

(A4) $$\begin{array}{l} M=\left(\begin{array}{cc} M_{xx} & M_{xy}\\ M_{yx} & M_{yy} \end{array}\right)\\ \;=\left(\begin{array}{cc} \int_{V}d^{2}\text{r}{\left(\Delta y\right)}^{2} & -\int_{V}d^{2}\text{r}\Delta x\Delta y\\ -\int_{V}d^{2}\text{r}\Delta x\Delta y & \int_{V}d^{2}\text{r}{\left(\Delta x\right)}^{2} \end{array}\right). \end{array}$$

Here, (Δ*x*, Δ*y*) is the position vector of each point from the center of mass of the plate (cell), *d*^2^**r** = *d*(Δ*x*)*d*(Δ*y*), and the integral $\underset{V}{\int }$ runs for the entire plate. This tensor **M** is calculated through the following formulae:

(A5) $$\begin{array}{l} M_{xx}=\frac{1}{12}\overset{I}{\underset{i=1}{\sum }}\left(\Delta {\text{r}}_{i}\times \Delta {\text{r}}_{i+1}\right)\left(\Delta y_{i}^{2}+\Delta y_{i}\Delta y_{i+1}+\Delta y_{i+1}^{2}\right), \end{array}$$

$$\begin{array}{l} M_{xy}=M_{yx}=-\frac{1}{24}\sum_{i=1}^{I}\left(\Delta {\text{r}}_{i}\times \Delta {\text{r}}_{i+1})[\Delta x_{i}(2\Delta y_{i}+\Delta y_{i+1}\right)\\ \;+\;\Delta x_{i+1}(\Delta y_{i}+2\Delta y_{i+1})], \end{array}$$

and

(A6) $$\begin{array}{l} M_{yy}=\frac{1}{12}\overset{I}{\underset{i=1}{\sum }}\left(\Delta {\text{r}}_{i}\times \Delta {\text{r}}_{i+1}\right)\left(\Delta x_{i}^{2}+\Delta x_{i}\Delta x_{i+1}+\Delta x_{i+1}^{2}\right), \end{array}$$

with the position vector Δ**r**_*i*_ = (Δ*x*_*i*_, Δ*y*_*i*_) of each vertex (*i*-th vertex) from the center of mass of the plate (cell). Here, the cell is assumed to consist of the vertices *i* = 1, 2, ⋯ , *I* designated in counter-clockwise order of the position seen from the cell's center, and the *i* = 1-th vertex is identified with the *i* = (*I*+1)-th vertex. The symbol × indicates a cross product in two dimensions, i.e., (*a*_1_, *a*_2_) × (*b*_1_, *b*_2_) = *a*_1_*b*_2_ − *b*_1_*a*_2_. From this analysis, we define the elliptical cell anisotropy *s*_α_ of α-th cell as the half difference of two eigenvalues of the tensor **M**, which is given by

(A7) $$\begin{array}{l} s_{\alpha }=\sqrt{{\left(M_{xx}-M_{yy}\right)}^{2}+4M_{xy}M_{yx}}. \end{array}$$

This quantity *s*_α_ is 0 when the plate is set to be a circle, and when the plate has an elliptically deformed shape from the circle, *s*_α_ has non-zero value. Therefore, we use *s*_α_ to quantify how the cell shape is elliptically deformed. Finally, the variable *s* is given as the average of *s*_α_ among the cells.

*Bond chirality c*(*t*). At each time step, we count the numbers *N*_cl_(*t*) and *N*_ccl_(*t*) of cell-cell junctions tilted clockwise and counterclockwise from the AP axis, respectively. We then define the bond chirality *c*(*t*) by *c*(*t*) ≡ *N*_cl_(*t*)/*N*_ccl_(*t*) − *N*_ccl_(*t*)/*N*_cl_(*t*). If the system has no chirality, *N*_cl_(*t*)/*N*_ccl_(*t*) = *N*_ccl_(*t*)/*N*_cl_(*t*) with the statistical average 〈·〉, and hence 〈*c*(*t*)〉 = 0.
