# Supplementary figures and images for: Wave Propagation of Junctional Remodeling in Collective Cell Movement of Epithelial Tissue: Numerical Simulation Study
Source: Front Cell Dev Biol. 2017 Jul 19;5:66. doi: 10.3389/fcell.2017.00066 (PMC5516087; doi:10.3389/fcell.2017.00066)

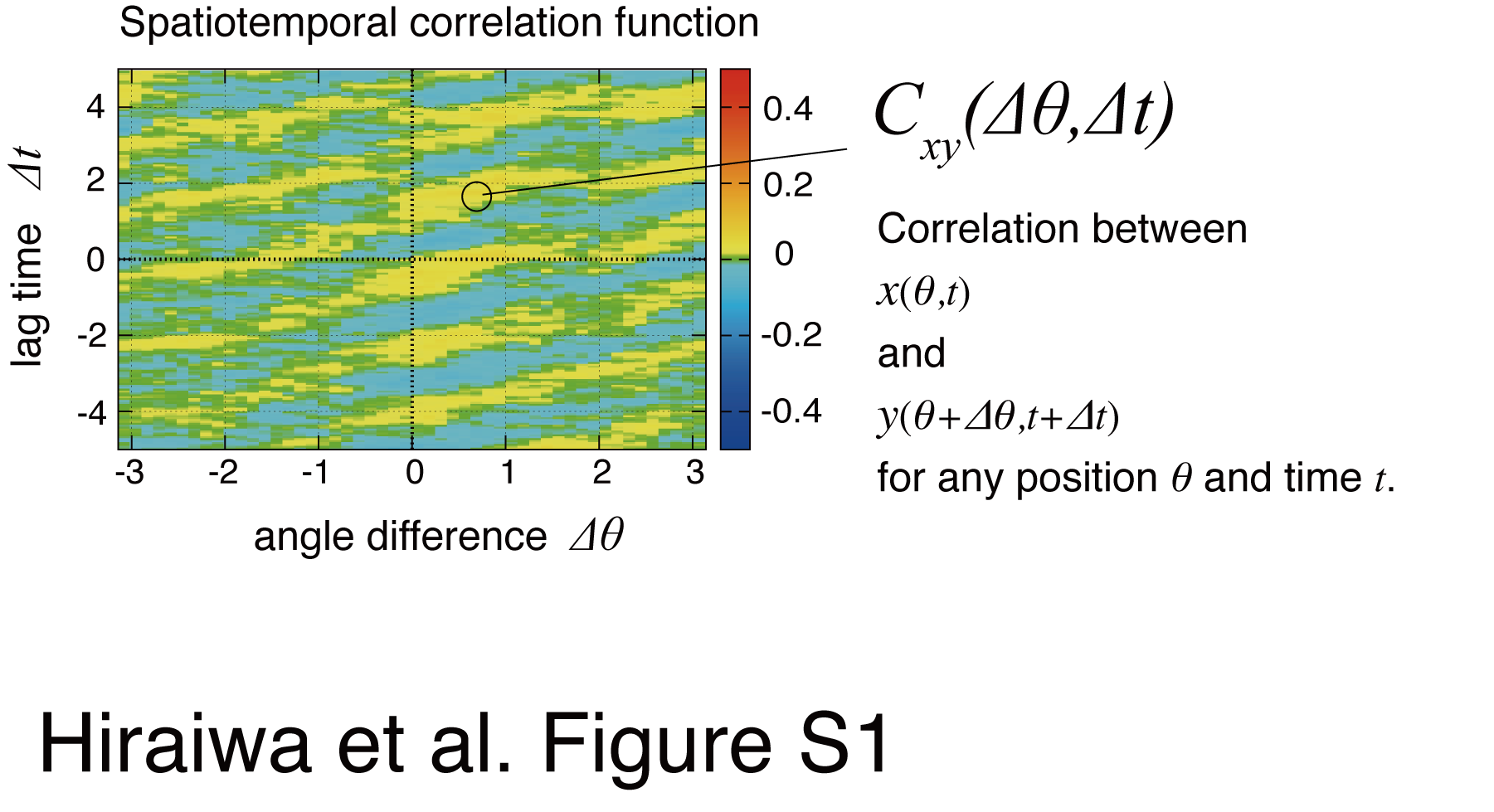

Supplement: Supplementary Figure 1 — Schematic explanation of spatiotemporal correlation function. [file Image1.TIF]
